# Supplementary material for: Intuitive or deliberative dishonesty: The effect of abstract versus concrete victim
Source: PLoS One. 2026 Jan 23;21(1):e0340083. doi: 10.1371/journal.pone.0340083 (PMC12829808; doi:10.1371/journal.pone.0340083)
Supplement: S1 Appendix — (DOCX) [file pone.0340083.s001.docx]

**S1 Appendix**

**Intuitive or Deliberative Dishonesty: The effect of abstract versus concrete victim**

Table S1. The pre- and post-game questions

| **Pre-game rule check questions** | | | | |
| --- | --- | --- | --- | --- |
| *1. If the toss results are 5-points, 2-points and 1-point in order. What points should you report?* | 1-point | 2-points | 5-points | I don’t know answer. |
| *2. If the toss results are 2-points, 6-points and 6-points. What points should you report?* | 1-point | 2-points | 6-points | I don’t know answer. |
| *3. If you report 5-points, how much payoff could you obtain?* | ¥1 RMB | ¥2 RMB | ¥5 RMB | I don’t know answer. |
| *4. If you report 5-points, how much payoff could the opponent obtain?* | ¥1 RMB | ¥2 RMB | ¥5 RMB | I don’t know answer. |
| **Post-game check questions** | | | | |
| *1. What do you know of the purpose of this game?* | (Short-answer Question) | | | |
| *2. Do you realize that cheat is allowed in the game?* | Yes, I realized | No, I don’t realize that |  |  |
| *3. How much does your task performance affect another subject's earnings* | five-point scale 1(not affect at all) to 5 (extremely effect) | | | |
| *4. How much you feel pressed for time in this game?* | five-point scale 1(no time pressured at all) to 5 (have much time pressured) | | | |

*Note.* Only participants those who passed the pre-game check questions could continue to the next page.
