# Supplementary material for: Intuitive or deliberative dishonesty: The effect of abstract versus concrete victim
Source: PLoS One. 2026 Jan 23;21(1):e0340083. doi: 10.1371/journal.pone.0340083 (PMC12829808; doi:10.1371/journal.pone.0340083)
Supplement: Attachment — Submitted filename: Response_Letter.docx [file pone.0340083.s003.docx]

**Response Letter**

Review PONE-D-25-29808

Title: Intuitive or Deliberative Dishonesty: The effect of abstract versus concrete victim

**Editor comment:**

Thank you for submitting your manuscript to PLOS ONE. After careful consideration, we feel that it has merit but does not fully meet PLOS ONE’s publication criteria as it currently stands. Therefore, we invite you to submit a revised version of the manuscript that addresses the points raised during the review process.

I have now received the reports from two reviewers with considerable knowledge and expertise in your topic domain (their detailed feedback appears below or as separate files). On the positive side, both the reviewers find your manuscript easy to follow and believe that you have interesting results based on a rigorous and preregistered empirical investigation. On the more critical side, however, they also raise a series of substantial concerns, which largely focus on lacking recruitment details, potential differential attrition across conditions, insufficient level of detail in the preregistered analyses, and clarity issues with respect to what was and was not preregistered. Moreover, they also note construct validity/confusion concerns, a failure to clearly discuss certain seemingly counterintuitive findings (e.g., more cheating in the concrete victim condition), and a lack of elaboration pertaining to whether, why, and how you believe that the study setting (online vs. real life) might have influenced your results, potentially due to lacking engagement among online participants (Baumeister et al., 2023). Further, the reviewers mention some uncited work which might bolster your theorizing and storyline.

Based on the constructive comments from the reviewers and my own reading of your paper, I am willing to move this manuscript into a second round of reviews. Given the magnitude of some of the issues identified by the reviewers, this will be a major revision, although a revision with a relatively clear path toward publication as long as you meticulously address all the substantive concerns flagged in the separate reviewer reports. Please make sure to reply to all comments made by the reviewers, incorporate needed changes in the manuscript, and then send an updated version of it along with your revision notes at your earliest convenience. Try to do this within the next two months. If you need additional time, feel free to let me know (at tobias.otterbring@uia.no) and I am happy to extend your revision window.

Reference

Baumeister, R. F., Tice, D. M., & Bushman, B. J. (2023). A review of multisite replication projects in social psychology: is it viable to sustain any confidence in social psychology’s knowledge base? Perspectives on Psychological Science, 18(4), 912-935.

**Response:** Thank you very much for your comment and for considering our manuscript for publication in *PLOS ONE*. We are grateful for the constructive comments provided by you and the reviewers. We truly appreciate your recognition of the strengths of our study, as well as the detailed feedback that has helped us to improve the manuscript substantially.

As will become clear in our response to the reviewers, we carefully considered all of the issues raised, including those concerning recruitment details (see page 7 in our revision draft), preregistration transparency (see pages 12 in our revision draft), potential attrition differences across conditions (see our response to Reviewer #2, Comment 2), discussion of counterintuitive findings (see pages 25-27 in our revision draft), and possible implications of conducting the study online (see pages 24-25 in our revision draft) etc. We have revised the manuscript accordingly and clarified these points in detail. We have also incorporated additional citations suggested by the reviewers to strengthen the theoretical framing.

In addition, we read Baumeister et al. (2023) and carefully considered the possible reasons for large-scale replication failures discussed by the authors, including that the original hypothesis was wrong; the hypothesis was not properly tested because of operational failure; the low engagement of participants; and bias toward failure. Although a prior multi-lab replication work has found that participants in online settings often display lower engagement and a diminished sense of “experimental realism,” which can weaken the effectiveness of manipulations and attenuate observed effects (Baumeister et al., 2023), it is also noteworthy that many online experiments have increasingly incorporated attention checks (Hauser & Schwarz, 2016; Zickfeld et al., 2025), comprehension questions (Parra et al., 2024), and other methodological safeguards, thereby gradually improving data quality (Douglas et al., 2023; Horton et al., 2011). Thus, we think that the assumption that lab participants are attentive and engaged but online participants is not that straightforward. Nevertheless, this issue underscores the importance of further refining experimental paradigms and strengthening experimental control in online settings to ensure the collection of higher-quality data. We have added a corresponding discussion of these issues in the revised manuscript (pls see pages 23-25*).*

In the text below, we provide a point-by-point response to all reviewer comments. We sincerely thank you and the reviewers for your constructive guidance and for giving us the opportunity to revise our work. We look forward to our revised manuscript moving to the second round of reviews.

**Reviewer #1:**

I have read your manuscript "Intuitive or Deliberative Dishonesty: The effect of abstract versus concrete victim" submitted to Plos One. Below is my review.
**Comment 1:** First, I applaud the fact that you conducted a preregistered well-powered study that aims to replicate earlier findings. This is surely a merit.

**Response:** Thank you for this positive comment.

**Comment 2:** Second, for the most part I think the paper is well written and easy to follow. You cite plenty of relevant research. Perhaps you want to look on Josh Greenes work on dual process morality where it is proposed that intuition leads to deontological decision making (following rules such as not harming) whereas deliberation leads to utilitarian decision making (maximizing good consequences). See Greene, J. D. (2008). The secret joke of Kant's soul. In W. Sinnott-Armstrong (Ed.), Moral psychology, Vol 3: The neuroscience of morality: Emotion, brain disorders, and development. (pp. 35-80). Cambridge: MIT Press. for an overview.

**Response:** Thank you very much for your kind feedback and the helpful suggestion regarding Greene’s work on dual process morality. We agree that his proposal—that intuitive processes tend to drive deontological decisions while deliberative processes support utilitarian reasoning—is highly influential and insightful. We did review Greene (2008) and found the perspective compelling. However, given that our study focuses specifically on (dis)honesty behavior in the context of abstract vs. concrete victims with time pressure rather than moral dilemma judgments per se, we felt that the dual-process morality framework was not the most fitting for our research aims. Still, we truly appreciate the suggestion and will consider referencing it in future related work.

I wonder whether you measure dishonestly or selfishness. In your study, lying is always selfish as it increases the participants’ payoff so dishonestly and selfishness are difficult to disentangle. Still, it is important to remember that both lies and truth telling can have both positive and negative (or no) consequences for both the person deciding whether to lie, and for the person being lied to. There are white lies, prosocial lies and altruistic lies. This should be highlighted and you should discuss the related literature investigating about whether prosociality is intuitive or deliberate. For instance Rand, D. G., Greene, J. D., & Nowak, M. A. (2012). Spontaneous giving and calculated greed [10.1038/nature11467]. Nature, 489(7416), 427-430. Tinghög, G., Andersson, D., Bonn, C., Böttiger, H., Josephson, C., Lundgren, G., Västfjäll, D., Kirchler, M., & Johannesson, M. (2013). Intuition and cooperation reconsidered. Nature, 498(7452), E1-E2.

**Response:** Thank you for this comment. Our research focuses on lying for self-profit. This is because the theoretical debates focus on situations where lying could bring benefits (Shalvi et al., 2012). However, we agree with the reviewer that there are other kinds of lies, such as white lies, prosocial lies and altruistic lies, and they are also worthy to examine. We have added one paragraph to discuss that future studies should also examine whether prosocial lies are intuitive or deliberative. See page 27:

“*Finally, it is important to recognize that dishonesty can take multiple forms, including self-serving, prosocial, and altruistic lies. The present research only focuses on lying for self-profits which is based on previous research. Future studies should examine whether prosocial lies is intuitive or deliberative. At the same time, we acknowledge that the broader literature emphasizes the diversity of lying motives and their links to (pro) social behavior (Rand et al., 2012; Tinghög et al., 2013). Future research would benefit from developing improved paradigms that examine prosocial and altruistic lies more directly, with a particular focus on the motivations underlying dishonesty and on how its consequences—whether positive, negative, or neutral—shape behavior.*”

I am not perfectly updated on the literature, but I feel that the fact that you did the die roll and reporting online rather than in real life can influence the believability of being able to lie without being detected. In the original die rolling studies, participants rolled a die in private and then reported the results on a separate paper (correct me if I am misremembering). This approach made it believable that one could cheat without being detected. In your online study, I feel that I would assume that you recorded the result of the die roll and could easily “prove that I lied” if you wanted to. You might not agree, but I feel you should discuss whether this rather big methodological difference could explain diverging results.

**Response:** Thank you for this valuable comment. We agree with the reviewer’s consideration that participants may think that they will be detected if they lied. In the typical laboratory version of this paradigm (see e.g., Van der Cruyssen et al., 2020), participants indeed rolled a die privately under a cup and observed the outcome through a small hole (visible only to themselves). They then reported the result, with higher reported outcomes yielding larger rewards (e.g., reporting a six earned €12). Because the outcome was known only to the participant, they had a credible opportunity to misreport without the risk of being caught. This procedure indeed allows for cheating without detection. Online implementations of this paradigm inevitably raise concerns about how anonymity is ensured. While no solution is perfect, several reasonable approaches have been proposed and adopted, including the method used in our study. Numerous related studies (e.g., Alfonso et al., 2022; Zickfeld et al., 2025) have successfully conducted this paradigm online, and meta-analyses suggest that, with appropriate controls, online results can be as reliable as those obtained in laboratory settings. In recent years, online experiments have increasingly incorporated methodological safeguards such as attention checks (Hauser & Schwarz, 2016; Zickfeld et al., 2025) and comprehension questions (Parra et al., 2024). Empirical evidence further supports that, when such measures are in place, online studies can achieve high levels of validity and reliability (Douglas et al., 2023; Horton et al., 2011). At the same time, it is important to note that online studies also offer a greater degree of anonymity and less social presence compared to laboratory settings. Previous studies suggest that the salience of interpersonal impact significantly influences the manifestation of dominant impulses in socially desirable or undesirable behavior (Gneezy, 2005; Jones, 1991; Köbis et al., 2019; Mok & De Cremer, 2018). Specifically, individuals exhibit a greater reluctance to inflict the identified victims compared to unidentified victims, primarily due to the heightened potential for inducing profound emotional distress by the former group (Kogut & Ritov, 2005; Milgram, 1965). This also highlights that online experiments offer many factors for further exploration. We call for future research to conduct comparative studies and develop new paradigms to better separate these factors. In the revised manuscript, we have expanded our Discussion about this issue (pls see page 23-25). We sincerely appreciate your suggestion, which helped us clarify this important aspect.

*“The first possibility is that methodological changes in the current study produced these results…*

*A further consideration is that the issue of lying without being detected is particularly salient in online experiments. In classic offline paradigms, participants could observe the die outcome privately and thus plausibly misreport without fear of being caught (Van der Cruyssen et al., 2020). By contrast, in our online setting, participants may have suspected that we recorded the actual roll, thereby enhancing the sense of exposure that cheating could be detected. Moreover, a prior multi-lab replication work has shown that participants in online often display lower engagement and a diminished sense of “experimental realism,” which can weaken the effectiveness of manipulations and attenuate observed effects (Baumeister et al., 2023). Online experiments inevitably raise concerns about being monitored/detected and feeling less engaged in the task. While no solution is perfect, several reasonable approaches have been proposed and adopted, including the methods used in our study. In recent years, online experiments have increasingly incorporated methodological safeguards such as attention checks (Hauser & Schwarz, 2016; Zickfeld et al., 2025) and comprehension questions (Parra, 2024). Empirical evidence further supports that, when such measures are in place, online studies can achieve high levels of validity and reliability (Douglas et al., 2023; Horton et al., 2011). However, in offline settings, participants may fear that their decisions are not anonymous, such that lying could threaten their public image of honesty toward the experimenters and thereby increase the psychological cost of dishonesty (Gerlach et al., 2019). By contrast, the heightened anonymity of online settings may reduce such concerns, making it easier for participants to cheat without inhibition. ”*

Relatedly, could it even be that what you measure is not dishonestly (lying in the belief that no one notices) but rather how much participants care about being caught lying?

**Response:** Thank you for raising this important point. We agree that, in principle, there is a distinction between measuring dishonesty per se and measuring how much participants care about being caught lying. Methodologically, our die-roll paradigm is designed to capture dishonesty by comparing self-reported outcomes with the actual probabilities of dice results. Participants always had an opportunity to increase their payoff by inflating their report, and since their individual rolls were private, the only way for them to gain extra points was through dishonesty. While we cannot completely rule out that some participants refrained from lying because they worried about being detected, the paradigm and incentive structure strongly support the interpretation that the primary behavior we measured was honesty versus dishonesty.

**Comment 3:** I like your method, but did you consider manipulating the die roll results rather than just observing them. In an online paradigm, this could easily be done I suppose and it would give you more control. Not saying that it must be done, but that it could be reflected upon.

**Response:** In online settings, there are indeed two ways to handle the die role outcome: manipulating it (Mazar et al., 2008) versus observing (Shalvi et al., 2011; Van der Cruyssen et al., 2020). We chose to control the outcome of the first die roll. Specifically, in the first roll, participants could only obtain numbers from 1 to 5, and the outcome “6” never appeared. This ensured that every participant had an opportunity to engage in dishonest reporting. Therefore, beyond controlling the first roll, subsequent dice outcomes were random. In the analyses, we used hierarchical regression, controlling for the first roll at the initial step. The results showed that smaller initial outcomes were associated with higher cheating rates and larger magnitudes of dishonesty. Future research could explore more complex ways of controlling dice outcomes to further disentangle these effects.

**Comment 4:** I believe the most surprising result is that people cheated more in the concrete victim conditions. This seems to be an effect in the opposite direction of what could be expected and against the identifiable victim effect. I strongly suggest that you discuss and try to make sense of this result. Not only a null effect but an effect in the opposite direction. Could it be that participants in the concrete victim conditions perceived it more like a game where the normative response was to “try to win” whereas participants in the abstract condition did not. Not sure, but in your version it seems like you want to hide this significant effect.

**Response:** Thank you very much for highlighting this issue and for suggesting a potential interpretation. We agree that this finding is both surprising and counterintuitive, running against what might be expected from the identifiable victim effect. The observed effect was statistically significant (*p* = .045) with a modest odds ratio (OR = 1.62, 95% CI [1.01, 2.59]), suggesting a statistically modest yet potentially meaningful difference. However, given the marginal p-value and wide confidence interval, the evidential value should be interpreted with caution. In the revised manuscript, we have expanded our discussion to openly consider possible explanations.

In particular, one possibility, as you suggested, is that participants in the concrete victim conditions may have perceived the task more as a competitive or game-like context, in which “trying to win” became a normatively acceptable response. In contrast, the abstract victim condition might not have evoked the same framing, leading to relatively less cheating. And inspired by your Comment 7, we considered that our results may reflect different moral orientations. As Moore et al. (2019) argue, many dilemmas can be seen as conflicts between benevolence (compassion for a specific individual; Mayer et al., 1995) and integrity (adherence to impartial rules; Mayer et al., 1995; McFal, 1987). Cheating in the abstract victim condition may thus reflect a failure of integrity, whereas cheating in the concrete victim condition may reflect a lack of benevolence. This perspective helps explain why dishonest behavior may be evaluated differently across conditions. We do not claim to have a definitive explanation, but we believe acknowledging and exploring these possibilities adds important nuance.

We are grateful for your suggestion, which helped us strengthen our discussion and provide a more transparent interpretation of this counterintuitive result. See Discussion page 26:

“*Another possibility, as suggested, is that participants in the concrete victim condition perceived the task more as a competitive or game-like context, in which “trying to win” became normatively acceptable. In contrast, the abstract victim condition may not have evoked the same framing, leading to relatively less cheating. Relatedly, Moore et al., (2019) highlighted that many everyday moral dilemmas can be understood as conflicts between benevolence (compassion for a specific individual; Mayer et al., 1995) and integrity (adherence to impartial rules, Mayer et al., 1995; McFall, 1987). Within this framework, cheating in the abstract victim condition may be interpreted as a failure of integrity (violating a general rule), whereas cheating in the concrete victim condition may reflect a lack of benevolence (disregarding the welfare of an identifiable other). This perspective helps clarify why the same dishonest behavior can be evaluated differently across conditions.”*

**Comment 5:** I do not really understand panel C on Figure 2. The x-axis display the two manipulated variables, but what does “score” on the Y-axis stand for?

**Response:** we apologize for the confusion caused by the figure. The y-axis refers to the distribution of the two post-question ratings (time-pressure self-report and harm self-report, both on a five-point scale). We have revised Figure 2c by adding the manipulation check questions in the figure to make it more interpretable on its own, without requiring reference to the main text. We have also revised the description in the main text accordingly. Thank you very much for pointing this out. Specifically, the caption now reads:

“*C) Violin plot of* ***rating scores (1 = not affected at all; to 5 = extremely affected)*** *on manipulation check questions for time-pressure and self-paced conditions, …*”.

**Comment 6:** The weak interaction effect of moral identity and time pressure condition is interesting but hard to make sense of. It makes sense that low moral ID and no time pressure leads to much cheating, but it does not makes much sense that time pressure leads to much cheating among those with high moral id. Or does it? I feel that you report results in a good way, but you could do more to at least offer possible reasons for the obtained results.

**Response:** We agree that the observed interaction effect is counterintuitive and goes against the idea of people with high moral identity having a ‘moral default’ towards honesty (Shalvi et al., 2025; Speer et al., 2020). One possible explanation is that time pressure may alter the way individuals with high moral identity process the situation. While high moral identity is generally expected to reduce dishonest behavior, under time pressure participants may rely more on intuitive or heuristic responses rather than deliberate moral reasoning. In the context of our task, we only give participants 13 seconds to complete the game and report the number, even those with high moral identity could default to this heuristic, resulting in more cheating under time pressure. In this sense, moral identity may buffer dishonesty only when sufficient time is available for reflection. When rushed, the motivational strength of moral identity may not fully translate into action.

In the revised manuscript, we have added an open discussion of these potential explanations, while emphasizing that more research is needed to clarify the mechanisms underlying this surprising interaction. Please see pages 26-27:

“*One possible explanation is that time pressure alters how individuals with high moral identity process the situation. Although moral identity is generally expected to show a ‘moral default’ (Shalvi et al., 2025; Speer et al., 2020), and reduce dishonest behavior, under time pressure participants may rely more on intuitive or heuristic responses rather than deliberate moral reasoning. In our task, the 13-second limit may have prompted even those with high moral identity to default to such heuristics, resulting in more cheating. Thus, moral identity may buffer dishonesty only when sufficient time is available for reflection. These findings should be interpreted with caution, and further research is needed to clarify the mechanisms underlying this counterintuitive interaction.*”

**Comment 7:** The study below seems relevant. I feel that cheating in the abstract victim condition is to act with low integrity (break a rule, and to act unfairly) wheres cheating in the concrete victim condition is to act with low benevolence.

Moore, A. K., Munguia Gomez, D. M., & Levine, E. E. (2019). Everyday dilemmas: New directions on the judgment and resolution of benevolence–integrity dilemmas. Social and Personality Psychology Compass, 13(7), e12472.

**Response:** Thank you for pointing out this relevant work and giving us this enlightening idea. We now cite Moore et al., (2019) in the Discussion section to strengthen our theoretical framing. Their framework highlights that many everyday moral dilemmas can be understood as conflicts between benevolence (acting out of compassion for a specific individual, see Mayer et al., 1995) and integrity (adhering to impartial rules and principles, see Mayer et al., 1995; McFall, 1987).

This distinction resonates with our findings: cheating in the abstract victim condition may be interpreted as a failure of integrity (violating a general rule), whereas cheating in the concrete victim condition may reflect a lack of benevolence (disregarding the welfare of an identifiable other). Incorporating this perspective clarifies why the same dishonest behavior may be evaluated differently across conditions, and it aligns with the broader literature on benevolence–integrity dilemmas. Now we add some sentences in the Discussion, please see page 25:

“*Another possibility, as suggested, is that participants in the concrete victim condition perceived the task more as a competitive or game-like context, in which “trying to win” became normatively acceptable. In contrast, the abstract victim condition may not have evoked the same framing, leading to relatively less cheating. Another study that inspired our thinking is Moore et al., (2019), which highlights that many everyday moral dilemmas can be understood as conflicts between benevolence (compassion for a specific individua, Mayer et al., 1995) and integrity (adherence to impartial rules, Mayer et al., 1995; McFall, 1987). Within this framework, cheating in the abstract victim condition may be interpreted as a failure of integrity (violating a general rule), whereas cheating in the concrete victim condition may reflect a lack of benevolence (disregarding the welfare of an identifiable other). This perspective helps clarify why the same dishonest behavior can be evaluated differently across conditions.”*

**Reviewer #2:**

This paper reports a preregistered experiment investigating whether time pressure (intuition) increases dishonesty (cheating behavior) when dishonesty harms an abstract (but not concrete) other. Results do not support this prediction, as there is no significant interaction effect between time pressure (vs. no time pressure) and harm type (abstract vs. concrete victim). However, there was a main effect of harm type such that participants cheated more when the victim was concrete than abstract, opposite of expectations. The experiment also explores individual traits.

The study addresses an interesting and relevant question and I think the paper is well-organized and clear and the conclusions appear warranted by the data. I hope my comments below can help improve the paper further.

**Response:** Thank you for this positive comment. We have carefully responded your comment point by point below.

**Comment 1:** Some more detail about how/where participants were recruited would be desirable (e.g. was it using a recruitment platform, social media, etc.).

**Response:** Thank you for this helpful comment. We have now provided more detail about participant recruitment in the revised manuscript. Please see page 7 at the current revised draft:

“*Specifically, participants were recruited from Hangzhou Normal University and Zhejiang University through both electronic and printed advertisements, and from Shenzhen University and Southwest University via online subject pools. Recruitment information was also disseminated via WeChat Moments. Interested individuals could access the study by scanning a QR code provided in the digital or printed posters, which directed them to the online game platform.”*

**Comment 2:** I am a bit puzzled by the results from the manipulation check for the harm type manipulation (concrete vs. abstract victim). The question asks: “How much does your task performance affect another subject’s earnings?” It seems to me that participants in the “abstract” condition should answer 1 (“not at all”) if they correctly understood the instructions, yet the mean rating is 2.31. Furthermore, one of the pre-game rule-check questions asks how much the opponent could obtain if the participant reports 5 points; given that participants in the abstract condition are not playing with an opponent, it is unclear what they should answer here. I wonder if there might be some selection effect because of this that affects the two conditions differently, as participants are screened out if they answer incorrectly. Also, how many participants in each condition failed the rule-check questions?

**Response:** Thank you very much for this thoughtful question. We agree that the manipulation check for the harm type manipulation may appear puzzling at first sight. As you noted, the item “How much does your task performance affect another subject’s earnings?” might be expected to yield a rating of “1 – not at all” in the abstract condition. However, the observed mean rating of 2.31 suggests that some participants interpreted the item in a broader sense, i.e., that their behavior still had consequences within the task framework even if no concrete opponent was paired with them. In other words, the abstract framing may have led participants to construe the manipulation check question less literally and more in terms of the experimental setting (such as time pressure or their individual traits), resulting in ratings above 1.

Regarding the pre-game rule-check question (“How much could the opponent obtain if the participant reports 5 points?”), we recognize that this could be somewhat confusing for participants in the abstract condition where no specific opponent exists. We regret that we were not able to track exactly how many participants may have withdrawn from the study at this stage. Because understanding the rules was crucial in an online setting, participants were allowed to answer the rule-check questions repeatedly until they provided the correct responses. Only after correctly answering all rule-check questions could participants proceed to the main task. Consequently, all participants who entered the game had correctly answered the rule-checks, and no one was excluded from the dataset on this basis. Still, we recognize that a small number of participants may have chosen to discontinue the study on their own if they found the rule-check questions confusing.

In addition, we carefully re-examined participants' feedback and comments for the experiment, and we did not receive any specific concerns or complaints related to this rule-check question. This gives us further confidence that the potential confusion did not systematically bias our final sample. We appreciate the reviewer's attention to this issue, as it highlights a limitation of our design that we had not sufficiently considered.

**Comment 3:** I would suggest reporting the full regression results from the main hypothesis test (section 3.1.2), considering that it is the main (and preregistered) hypothesis test.

**Response:** Thank you for this helpful suggestion. We agree that, given this is our main and preregistered hypothesis test, it is important to report the full regression results. In the revised manuscript, we have now expanded Section 3.1.2 to present not only the overall model fit statistics but also the coefficients, standard errors, Wald tests, p-values, odds ratios, and 95% confidence intervals for each predictor. Specifically, we report the results for time pressure, harm type, and their interaction in detail. We believe that including these full regression results in the main text enhances the transparency and clarity of our reporting. See pages 14-15

“*Further inspections showed that participants in the time-pressure condition (M = 0.27, SD = 0.44) did not lead to more cheating than participants in the self-paced condition (M = 0.25, SD = 0.43), B = 0.15, SE = 0.25, Wald (1) = 0.38, p = .535, OR = 1.17, 95% C.I. = [0.72, 1.90]. Interestingly, we found participants cheated more in the concrete harm condition (M = 0.30, SD = 0.46) than abstract harm condition (M = 0.22, SD = 0.41), B = 0.48, SE = 0.24, Wald (1) = 4.02, p = .045, OR = 1.62, 95% C.I. = [1.01, 2.59]. Importantly, inconsistent with our prediction, the interaction between time pressure and harm types was also not significant, B = – 0.08, SE = 0.33, Wald (1) = 0.06, p = .812, OR = 0.92, 95% C.I. = [0.48, 1.78].*”

**Comment 4:** In some places it could be made clearer what was and what was not preregistered among the analyses. In particular, it could be mentioned in the analysis section that the use of Bayes factors was not preregistered.

**Response:** we appreciate this comment. As suggested, we have clarified in the revised manuscript which analyses were preregistered, and which were not. Please see pages 13 for the details.

**“*2.4. Data analysis***

Data were coded and analyzed using IBM SPSS Statistics 29 (IBM Corp., Armonk, NY, USA) with a significance set at *p* < 0.05. Below, we distinguish preregistered from non-preregistered exploratory analyses.

*2.4.1 Preregistered analysis*

According to our preregistration… affect another participant’s earnings.

*2.4.2 Non-preregistered analyses*

Beyond the preregistered plan… Bayes factors enhance the transparency and informativeness of our findings.”

**Comment 5:** Figure 2c (manipulation checks) could be modified slightly to improve interpretability on its own (without having to read the text). E.g. add the manipulation check questions into the figure as subheadings or similar.

**Response:** We apologize for the lack of clarity in the original figure, and thank you very much for this helpful suggestion. Following your advice, we have revised Figure 2C by adding the manipulation check questions in the figure to make it more interpretable on its own, without requiring reference to the main text.

**Some minor comments:**

1. Look over the grammar in this sentence: ”Other experimental works have similarly revealed … will increase self-serving dishonesty.” (p 3-4)

2. “cognitively impaired” (p 4) has a different connotation than what I think the authors intend – change to “cognitively depleted” or “temporarily cognitively impaired”?

3. Preregistrated (p 5) -> preregistered

4. Mofied -> modified (p 5)

5. Pista et al./Pistea et al. -> Pitesa et al. (p 5)

6. ”Exploratory” -> ”As exploratory analyses” or similar (p 6)

7. present study -> the present study (p 6)

8. preregistratrion study -> preregistered study (p 23)

9. P. 11: “t-tests assessed the manipulations' effects on time pressure and harm types” – I believe this refers to the manipulation checks? I suggest a slight rephrasing for clarity, e.g. “t-tests assessed the manipulations’ effects on participants’ subjective experience of time pressure and whether participants understood that their results in the game would (or would not) affect another participant’s earnings” (or whatever the authors find more appropriate).

**Response:** Thank you very much for these detailed and helpful comments on wording and grammar. We have carefully revised the manuscript accordingly.
